# Suicide Risk Screening in Jails: Protocol for a Pilot Study Leveraging the Mental Health Research Network Algorithm and Health Care Data

**DOI:** 10.2196/68517

**Published:** 2025-06-25

**Authors:** Erin B Comartin, Grant Victor, Athena Kheibari, Brian K Ahmedani, Bethany Hedden-Clayton, Richard N Jones, Ted R Miller, Jennifer E Johnson, Lauren M Weinstock, Sheryl Kubiak

**Affiliations:** 1 School of Social Work Wayne State University Detroit, MI United States; 2 School of Social Work Rutgers University New Brunswick, NJ United States; 3 Center for Health Policy & Health Services Research Henry Ford Health Systems Detroit, MI United States; 4 Center for Behavioral Health & Justice Wayne State University Detroit, MI United States; 5 Department of Psychiatry and Human Behavior Warren Alpert Medical School Brown University Providence, RI United States; 6 Pacific Institute For Research and Evaluation Calverton, NY United States; 7 Charles Stewart Mott Department of Public Health Michigan State University Flint, MI United States

**Keywords:** suicide, suicide prevention, health risk behaviors, machine learning, jails

## Abstract

**Background:**

Suicide in local jails occurs at a higher rate than in the general population, requiring improvements to risk screening methods. Current suicide risk screening practices in jails are insufficient: They are commonly not conducted using validated screening instruments, not collected by clinically trained professionals, and unlikely to capture honest responses due to the chaotic nature of booking areas. Therefore, new technologies could improve such practices. Several studies have indicated that machine learning (ML) models considerably improve accuracy and have positive predictive value in detecting suicide risk compared with practice as usual (PAU). This study will use administrative data and ML modeling to improve suicide risk detection at jail booking.

**Objective:**

This study is primarily focused on gathering preliminary information about the feasibility and practicality of using administrative data and ML modeling for suicide risk detection but also incorporates elements of hypothesis testing pertaining to clinical outcomes.

**Methods:**

The study uniquely contributes to our understanding of suicide risk by further validating an existing ML model developed and previously validated by the Mental Health Research Network using Medicaid outpatient health care claims data. This validation uses complete claims data on a sample of approximately 6000 individuals booked into 2 diverse jails in a midwestern state. This model validation uses 313 unique demographic and clinical characteristics from 5 years of historical health care data. It detects suicide risk in jails and postrelease by using merged jail, Medicaid, and vital records data. The study will use jail administrative data for September 1, 2021, through February 28, 2022; Medicaid records data for September 1, 2016, through March 31, 2023; and vital records data for March 1, 2022, through March 31, 2023.

**Results:**

First, the algorithm will be validated on the data gathered for the jail sample using the C-statistic and area under the receiver operating characteristic curve. Second, the resulting model will be compared with the jails’ suicide identification PAU to assess risk and detection of identified suicide attempts and deaths from intake through 120 days and 13 months after jail release. The funding timeline for this project is August 1, 2022, through July 31, 2025. The algorithm’s predictions and actual event incidence will be linked and validated in the spring of 2025, with results ready for publication in the fall of 2025.

**Conclusions:**

The study will also investigate implementation factors, such as feasibility, acceptability, and appropriateness, to optimize jail uptake. Interview data on the implementation factors will be gathered in the summer of 2025, with expected dissemination in 2026. We hypothesize that a combination of intake screening PAU and the ML model will be the optimal approach, in that the combination will be more accurate and can have practical application in this context.

**International Registered Report Identifier (IRRID):**

DERR1-10.2196/68517

## Introduction

### Suicide Risk in Jails and Standard Screening Procedures

The most recent available data indicate that suicide is the leading cause of death among those incarcerated in jails, and the suicide rate among those detained in jails is approximately 4 times greater than those in the public [1,2]. Long-term trends suggest that suicide has been the leading cause of in-jail deaths from 2006 to 2016 following steady increases in suicide mortality since 2000, and it accounted for nearly 30% of all in-jail deaths in 2019 [3]. A period of incarceration is also associated with suicide mortality postrelease, as evidence indicates that the risk of suicide doubles after jail release [4].

Few jails effectively screen for suicide risk at jail booking. Due to the fast-paced booking process, most jails use nonstandardized, self-report, or single-item measures that can lack cultural sensitivity or are inappropriate for this setting [5,6]. Given that many jail booking areas are not private, individuals may be less forthcoming in reporting suicidal ideation to jail staff. Current suicide risk identification practices are insufficient given the impracticability of having clinically trained professionals conduct adequate assessments at booking. These deficiencies in suicide risk screening in jail settings exemplify the need to improve risk detection. Improved suicide risk identification could reduce the persistent missingness of behavioral health data in jail facilities [7] while mitigating the adverse impacts that suicide has on detainees, families, jail staff, and the community [8].

### Machine Learning for Suicide Risk Screening

Machine learning is the basis for suicide risk identification models. Several studies have indicated that machine learning models considerably improve accuracy and have positive predictive value in detecting risk of suicide compared with practice as usual (PAU) [9-11]. Suicide risk identification via machine learning detection has already been applied in health care systems [9,12], psychiatric care [11], and cross-sectional [13,14] and longitudinal [15,16] study designs focused on suicide risk identification, with earlier estimates of suicide attempt and death accuracy ranging from the high 0.80s to low 0.90s, meaning predictions are accurate nearly 90% of the time [10]. Further, a recent systematic review of machine learning that was applied to predict suicide among psychiatric patients reported that predictive accuracy was reliably above 0.70—with 14 studies between 0.80 and 0.90 and 11 studies above 0.90 [11]. The systematic review by Pigoni et al [11] and meta-analysis by Ehtemam et al [9] demonstrated that the strongest associations of suicide included prior suicide attempts and behaviors [17,18] and diagnosis of major depressive disorder or borderline personality disorder, as well as historical psychiatric hospitalizations [11].

The Mental Health Research Network (MHRN) developed and validated a suicide risk identification machine learning model that detects suicide attempt and death risk based on historical health and insurer (including Medicaid) data [19]. Among a general population sample, the MHRN model has shown effectiveness at identifying increased risk for suicide attempts and deaths using general and behavioral health records and claims data across 7 health care systems [19] and from claims data alone [20]. The MHRN model generates a suicide risk score that can be used to alert health personnel to the possibility of heightened suicide risk and prompt further screening and service provision.

The MHRN model gathers 313 unique factors such as demographic and clinical characteristics from 5 years of historical health care data. The MHRN model outperformed other suicide screens and machine learning models with a C-statistic, area under the receiver operating characteristic (ROC) curve, of 0.853 (95% CI 0.849-0.857) compared with the Recovery Engagement and Coordination for Health-Veterans Enhanced Treatment (REACH VET) model, which has a C-statistic of 0.761 (95% CI 0.751-0.771) [21], with the top 5% of risk scores identifying over 43% of suicide attempts or deaths 90 days following a health care visit. The MHRN model includes a comprehensive set of clinical indicators, is intended to be used at the point of encounter, and shows promising results for improved suicide risk identification among a sample of the general population. A similar identification process for incarcerated individuals, available at jail booking, could help detect those at risk for in-jail and postrelease suicide attempts and deaths. Because there is no standardized process for identification of suicide risk across jails, an objective data-driven risk indicator would greatly enhance such screening.

### Study Aims

Our protocol includes 3 interrelated aims. The first unique contribution this study makes to suicide risk identification is that it validates the MHRN model using retrospective data from approximately 6000 individuals booked into 2 geographically and demographically diverse jails in a midwestern state to assess model generalizability. Jail administration data will be linked with historic (ie, prebooking) Medicaid claims data so the MHRN model can generate suicide risk scores. Since Medicaid is the insurer for 73% of the jail population in the study’s state [22], most of the jail population will be included in the analysis. The second aim seeks to compare the accuracy of the MHRN flag at identifying risk of suicide attempt and death to current suicide risk identification PAU over 120 days and 13 months from initial jail booking among individuals who have been released from jail. This involves merging jail records and prospective Medicaid, pharmacy, and vital records data to examine if the MHRN model improved suicide risk detection in jails and postrelease as compared with the jails’ PAU. The research team hypothesizes that the MHRN model will be validated as an effective screening tool to detect risk for suicide attempts and deaths as evidenced by a C-statistic (area under the ROC curve) of at least 0.75 and a positive predictive value that exceeds the base rate of suicide attempts and deaths through 13 months postbooking [23]. The pilot testing is included in the third aim, which will evaluate implementation outcomes related to integrating the MHRN model in jails.

This pilot project benefits from having a substantive sample size that will allow for an evaluation of the implementation of the MHRN model in jails and to conduct hypothesis testing on select clinical outcomes. For instance, if the MHRN model improves jails’ PAU, increased opportunities to connect individuals at risk of suicide with interventions to reduce suicide attempts and deaths in jail and postrelease will be created. The research team hypothesizes that more individuals with subsequent suicide attempts or deaths will be identified by the model or a combination of the model and screening versus screening only.

## Methods

### Overview

Two participating jails will provide data on booking and suicide risk identification PAU results for their jail population over a 6-month period (September 1, 2021, through February 28, 2022). To assess suicide risk, the MHRN model will be applied to 5 years of Medicaid encounter data prior to jail booking (September 1, 2016, through August 31, 2021). We will compare the accuracy of the MHRN model, the jail’s screening PAU at identifying suicide attempts, and deaths in jail and over 13 months after jail release using Medicaid and pharmacy data for suicide attempts and vital records data for suicide deaths (both sources [Medicaid and pharmacy data] as well as vital records data: March 1, 2022, through March 31, 2023).

### Ethical Considerations

The study was approved by the Wayne State University institutional review board (#22064717). Informed consent was waived because there is no more than minimal risk to the participants, participation in the study will not adversely affect their rights and welfare beyond data privacy and confidentiality, and the study could not practicably be carried out without the waiver. Data use agreements were legally established between the research team and entities providing personal identifiable information (PII). The final data set will be linked together using PII, and once verified, all PII will be destroyed. All data will be maintained on an encrypted and secure cloud server (Microsoft OneDrive) that only allows access to a small number of research team members.

### Analytic Focus

National Center for Health and Justice Integration for Suicide Prevention (NCHATS) research studies are grounded in application of data analytic approaches to identify individuals at risk for suicide across different intercepts of the sequential intercept model [24]. This project focuses on suicide risk detection during incarceration, whereas other NCHATS projects focus on suicide risk detection and prevention before and after incarceration.

### Jail County Sites

Two county jails are participating in the study. Jails were selected based on their varying geographic settings and jail populations. County A is a metropolitan-sized county on the west side of the state with a population of approximately 670,000 people. The jail has 1285 beds and booked 24,000 individuals in 2019. County B, on the east side of the state, is an urban county, with over 370,000 people. The jail had 404 beds and 8300 bookings in 2019. These jails vary in screening methods for suicide risk at jail booking, which reflects variability nationally. County A uses several scripted questions about current and past suicide ideation or attempts, and County B uses several scripted questions and a truncated version of the Columbia Suicide Severity Rating Scale [25].

The 2 jail populations vary demographically. County A is just under one-half White (46.5%), while County B is less White (39.4%). County A has a smaller proportion of men (71.4%) than County B (76.4%). Both jail populations are similar in age, with County A having 51.3% of people 31 years or older and County B with 51.5%. Medicaid is the insurer for 96% of people in jail in County A and 78% of people in jail in County B. Prior studies show varying rates of mental health needs, assessed by a modified cutoff score on the Kessler-6 [26], with County A (21.5%) reporting higher proportions than County B (16.8%). According to the Bureau of Justice Statistics [27], the average jail length of stay nationally was 33 days in 2021, an increase of 5 days from the previous year (28 days in 2020).

### Data Sources and Assessments

#### Jail Data

Jail data will include admission and discharge dates and jail medical records, including results from the suicide identification PAU (positive or negative), intervention or referral related to suicide risk, veteran status, and information on suicide attempts or deaths while in jail. To determine if a death occurred within the jail, the research team will use jail admissions and discharge dates and date of death from vital records data to determine if the death occurred inside the facility. Self-injury vital record deaths that occur within 24 hours of jail discharge will be considered an in-custody suicide, accounting for jail to hospital transfer.

#### Community Health and Death Data

So that individuals can be matched across data sets, the research team will provide identifiers to the state’s Department of Health and Human Services, which oversees Medicaid, pharmacy, and vital records data. To account for the average time spent in jail, the postrelease period will be extended to 120 days and 13 months, as opposed to 90 days and 12 months [19]. Physical and behavioral health Medicaid encounter data and pharmacy data include date of service between September 1, 2016, and March 31, 2023; current procedural terminology code; procedure code; diagnosis-related groups code; national drug code; medication class, generic name, dose; medication provider; medication days of supply and fill dates; member ID type; quantity of service and length of stay; transactional control number; national provider ID; diagnosis code and sequence; encounter or provider type; zip code; census tract; and member-months. Identifiers such as date of birth and first and last name will be needed for record linkage purposes. The research team will use the MHRN model definition for suicide attempt, which includes International Classification of Diseases, Tenth Revision (ICD-10) diagnosis of suicide attempt (example codes: suicide attempt [T14.91]; self-inflicted injury/poisoning due to asphyxiation [T71.X]) as measured using Medicaid encounter data. Vital records data requested consists of the following variables recorded from March 1, 2022, through March 31, 2023: state and county of occurrence; state, county, and zip code of residence; census tract; date of death; age; sex; race; marital status; autopsy; underlying cause of death code (Code800); related cause of death; if the case was referred to the medical examiner and the certifier of the death record; place and manner of death; and the decedent’s first and last name and birth date. An ICD-10 diagnosis of self-inflicted injury (codes X60-X84) or injury/poisoning with undetermined intent (codes Y10-Y34) will be used to capture suicide deaths from the vital records data. To ensure the accuracy of the match and determine missingness, the research team will conduct intense data cleaning on these sources, removing individuals not found in Medicaid data. If individuals are not found in the vital records data, it is assumed death did not occur since these are official records. Table 1 outlines the key identification methods and post-year outcomes, along with corresponding data sources.

**Table 1:** Key measures and data sources.

Key measures		Source
**Suicide risk identification method**		
	Model (5th-10th percentile groups)	Medicaid data
	Practice as usual (PAU)	Jail data
**Suicide behavior outcomes (1 year after jail)**		
	Suicide deaths	Vital records
	Suicide attempts	Medicaid data, jail incident reports

## Results

### Study Status

This study is funded by the National Institute of Mental Health to NCHATS. The funding timeline for this project is August 1, 2022, through July 31, 2025. The algorithm’s predictions and actual event incidence will be linked and validated in the spring of 2025, with results ready for publication in the fall of 2025.

### Quantitative Data Analysis

This study will determine if the model is successful at identifying suicide attempts and deaths during and after jail detention. Assessment of drops in predicted probability for every 1% increment between 5% and 10% will inform the creation of a cutoff percentile for this population. Univariate analyses for identifying suicide outcomes from intake through 120 days and 13 months after jail release will include 3 suicide risk identification methods: (1) model only, (2) PAU only, and (3) model and PAU. Risk identification methods will be compared within each jail separately and in both jails together. Frequencies of suicide risk identification will be summarized, and associations between identification method and suicide outcomes will be examined using chi-squared analyses. Predictive validity of risk identification methods for assessing suicide attempts and deaths (separately and combined) will be calculated using ordinal logistic regression models. Adjusted odds ratios and their 95% confidence intervals will be estimated. Nonindependence of observations within jails will be considered using robust standard errors that account for the intracluster correlation. A secondary set of analyses will also include suicide attempts reported within jails. Moderation analyses will explore whether MHRN and PAU accuracy vary by sex; race or ethnicity; past suicide attempt; past behavioral health treatment; county; the Area Deprivation Index, which reflects income, education, employment, and housing quality; Mental Health Professional Shortage Area score; and per capita incarceration.

**Figure 1:**
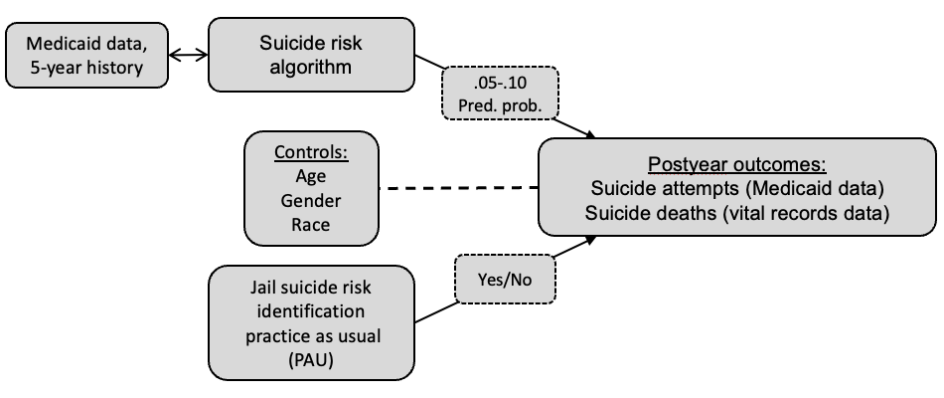
Analysis model. Pred. prob.: predictive probability.

The model will be validated using SAS (SAS Institute). SAS will return the predicted probabilities and percentile groupings across the sample. The same percentile groups used in the MRHN study will be used for analysis in this pilot (5th percentile), with expansion to higher test levels (10th percentile) to allow state-level partners and jail staff to assess any gains that could be made at each 1% interval in probable scores (Figure 1) [19].

### Cost Analysis

To facilitate replication, the study team will track the hours spent negotiating access to the linkable data sets, programming the linkage and scoring, and developing a user interface appropriate to the jail setting. Costs for these 3 tasks will be reported and include salaries, fringe benefits, overhead, and any other direct costs (eg, for data storage).

### Administrator Interviews

Through semistructured interviews with state-level administrators involved in the health and jail data linkage study and jail staff across both jail sites, the research team will assess the facilitators and barriers of utilizing the MHRN model as a suicide risk flag. These interviews will assess the jails’ information technology and software capacity for and barriers to embedding the MHRN model at jail booking as well as institutional policies, procedures, and practices that hinder or facilitate future implementation of the MHRN model in jails. Guided by the Institute for Healthcare Improvement Framework for Going to Full Scale [28], broad and jail-specific implementation factors of acceptability, feasibility, and appropriateness will be assessed per the Acceptability of Intervention Measure, Intervention Appropriateness Measure, and Feasibility of Intervention Measure [29]. This information will be used to design potential implementation approaches for future testing.

### Qualitative Analyses

All interviews will be audio-recorded and transcribed and analyzed by multiple coders using a grounded theory approach to understand facilitators and barriers to implementation through a content-analytic matrix and by jail staff role through role-ordered matrix [30,31]. Findings will be used in the creation of a data integration tool kit that will provide summarized recommendations to state and jail partners on what is needed to efficiently and effectively implement the MHRN model into the jails’ systems. The tool kit will also include recommended actions needed to respond to suicide risk in jails.  

## Discussion

### Overview

This article describes a research protocol for validating a community suicide risk identification machine learning model with jail populations by comparing the model with other validated instruments and screening practices to improve data surveillance and treatment linkage for a highly vulnerable population. By integrating data across general health, behavioral health, and criminal-legal systems, providers could leverage an existing high-performing suicide risk model to detect risk of suicide in a novel jail population [19]. Using data linkage to ensure access to critical and life-saving information at jail booking would greatly increase the probability of appropriate intervention. The implications of improved suicide risk detection for clinical and community impacts would be clear: (1) greater justification for enhancing mental health care in carceral settings, (2) reduced harm on communities by preventing suicide exposure (eg, community impact on jail staff, other incarcerated individuals, and families), and (3) improved efficiency for processing behavioral health data and reduced likelihood for missing key information. These benefits would contribute to the potential scalability of implementing suicide risk identification model technology. Although this is a complicated endeavor, marked by information technology infrastructure and data sharing legality challenges, authors and state-level officials in the study state are engaged in ongoing efforts to better integrate community and jail health data. Key considerations for these ongoing efforts to link data sources and apply the algorithm are the data privacy and security measures needed to protect sensitive behavioral health and criminal-legal data from this vulnerable population. The highest standards of protection will be adopted to assure that no future harm from data breaches occurs (ie, identity theft, financial losses).

More accurate and efficient ways of identifying vulnerable individuals at high risk of suicide and rapidly connecting them to treatment are needed. This project attempts to fill this gap using an automated, algorithmic screening tool that has demonstrated effectiveness in prior medical settings [19]. Should the MHRN model prove to be a stronger alternative to jails’ PAU or to improve accuracy when combined with the PAU, the next steps will be to understand how it might be replicated in other areas. Project implementation analyses will identify implementation facilitators and develop strategies to address potential implementation barriers, such as limited medical health care in carceral settings [32], wide-ranging variations in service quality in carceral settings [33], and mental health stigma [34]. We anticipate that additional potential barriers to implementation identified in other research, such as ethical, technological (eg, data quality, data access), liability and regulatory (eg, responsibility for outcomes and decisions), and workforce (eg, training and willingness) [35], may be relevant to this study.

Although this study is intended to validate the MHRN model in a new clinical population, there are limitations to consider. Only 2 jails from one midwestern state will be used for validation: one in a metropolitan area and the other in an urban area. Given that no jail in a rural community will be represented in the validation, the results may not be generalizable to that geographic context. Rural validation would be desirable if the urban validation is successful. In addition, limitations exist in the data sources used for our outcome variables of suicide attempt and death. Like in the initial validation, this validation restricted its nonfatal event detection to Medicaid claims data; thus, attempts were missed during periods when the person was uninsured, was insured by another payer, or received care that was not billed (eg, from a community mental health center or the Veterans Administration). Despite these limitations, this study will close the main gap in MHRN model validation in the Medicaid population. If the model validates there, the most pressing validation need remaining is to assess MHRN accuracy in a higher income group of privately insured people.

### Conclusion

There is a growing interest in using machine learning to detect suicide risk, and through this study, this promising methodology will be applied to an incarcerated population. Machine learning to detect suicide risk among incarcerated individuals holds promise for improving the identification and prevention of suicide. However, it is important to rigorously validate machine learning algorithms and carefully consider the potential advantages of use before they are widely implemented. This project will (1) accomplish this important next step, validating the models overall and by sex, race and ethnicity, and other important moderators, and (2) explore implementation strategies to be tested in future trials, should tested algorithms prove effective.

## Abbreviations

MHRN: Mental Health Research Network
NCHATS: National Center for Health and Justice Integration for Suicide Prevention
PAU: practice as usual
PII: personal identifiable information
REACH-VET: Recovery Engagement and Coordination for Health-Veterans Enhanced Treatment
ROC: receiver operating characteristic

## Data Availability

The data sets generated during this study are not publicly available due to privacy and confidentiality concerns but are available from the author on reasonable request.
